# 906. Characteristics of Chronic Hepatitis B Patients with Severe Outcomes in a Large Integrated Healthcare System - 2008-2019

**DOI:** 10.1093/ofid/ofab466.1101

**Published:** 2021-12-04

**Authors:** Ana Florea, Prabhu Gounder, Amandeep Sahota, Katherine J Pak, Vennis Hong, Theresa M Im, Sara Tartof

**Affiliations:** 1 Kaiser Permanente Southern California, Pasadena, CA; 2 Los Angeles County DPH, Los Angeles, CA; 3 Kaiser Pemanente, Pasadena, CA; 4 Kaiser Permanente, El Monte, CA; 5 Kaiser Permanente Southern California Department of Research & Evaluation, Pasadena, CA

## Abstract

**Background:**

It is estimated that there are 1.59 million cases of chronic hepatitis B virus (HBV) infection (CHB) in the United States. HBV infection is highest among men and non-Hispanic Asian adults. CHB can lead to liver damage, cirrhosis, hepatocellular carcinoma, or death. However, the population that is most likely to develop severe outcomes is not as well-defined.

**Methods:**

We evaluated electronic health record data from Kaiser Permanente Southern California adult members from 2008-2019 with at least 1 year of continuous membership, and with 2 successive, positive HBV lab results (HBV DNA, or HBsAg, or HBeAg) at least 6 months apart (indicative of CHB). Severe outcomes included incident hepatic decompensation, hepatocellular carcinoma (HCC), liver transplant and death, and prevalent and incident liver cirrhosis. For each outcome, we estimated the distribution of characteristics including age, sex, race/ethnicity, and lab values (alanine aminotransferase [ALT], alpha-fetoprotein [AFP], MELD score).

**Results:**

Our final study population included 5,427 CHB-diagnosed patients with 411 (7.6%) cases of liver cirrhosis, 123 (2.3%) of hepatic decompensation, 65 (1.2%) of HCC, 8 (0.1%) of liver transplant, and 164 (3.0%) deaths. Compared to the total cohort, those who developed severe outcomes were older (median age for each outcome >50 years vs. 47 years in total CHB population). Among those with severe outcomes, the majority were male ( >56%) and Asian. Diabetes was more prevalent in patients with hepatic decompensation, HCC, and death versus the entire cohort (25% vs. 8%, respectively, P< 0.0001), and twice as prevalent among those with cirrhosis. All severe outcomes were associated with >2 x upper limit of normal ALT levels.

**Conclusion:**

The characteristics of those with severe outcomes were consistent with those of overall CHB, although there was a 2-3 times higher prevalence of diabetes in those with severe outcomes. Identifying characteristics that are more prevalent in those with severe outcomes can help inform screening and management of CHB.

**Disclosures:**

**Ana Florea, PhD MPH**, **Gilead Inc.** (Grant/Research Support) **Prabhu Gounder, MD**, **Gilead Inc.** (Grant/Research Support) **Amandeep Sahota, MD, MS**, **Gilead Inc** (Grant/Research Support) **Katherine J. Pak, MS**, **Gilead** (Grant/Research Support) **Vennis Hong, MPH**, **Gilead Inc.** (Research Grant or Support) **Theresa M. Im, MPH**, **Gilead Inc.** (Grant/Research Support) **Sara Tartof, PhD**, **Gilead** (Grant/Research Support, Scientific Research Study Investigator)

